# Conformational and Thermal Stability Improvements for the Large-Scale Production of Yeast-Derived Rabbit Hemorrhagic Disease Virus-Like Particles as Multipurpose Vaccine

**DOI:** 10.1371/journal.pone.0056417

**Published:** 2013-02-27

**Authors:** Erlinda Fernández, Jorge R. Toledo, Lídice Méndez, Nemecio González, Francisco Parra, José M. Martín-Alonso, Miladys Limonta, Kosara Sánchez, Ania Cabrales, Mario P. Estrada, Alina Rodríguez-Mallón, Omar Farnós

**Affiliations:** 1 Division of Animal Biotechnology, Center for Genetic Engineering and Biotechnology, Havana, Cuba; 2 Department of Physiopathology, Faculty of Biological Sciences, University of Concepción, Concepción, Chile; 3 Technological Development Department, Center for Genetic Engineering and Biotechnology of Camaguey, Camaguey, Cuba; 4 Department of Biochemistry and Molecular Biology, Instituto Universitario de Biotecnología de Asturias, University of Oviedo, Oviedo, Spain; 5 Division of Technological Development, Center for Genetic Engineering and Biotechnology, Havana, Cuba; 6 Division of Development of Formulations, Center for Genetic Engineering and Biotechnology, Havana, Cuba; 7 Peptide Synthesis Department, Center for Genetic Engineering and Biotechnology, Havana, Cuba; Instituto Butantan, Brazil

## Abstract

Recombinant virus-like particles (VLP) antigenically similar to rabbit hemorrhagic disease virus (RHDV) were recently expressed at high levels inside *Pichia pastoris* cells. Based on the potential of RHDV VLP as platform for diverse vaccination purposes we undertook the design, development and scale-up of a production process. Conformational and stability issues were addressed to improve process control and optimization. Analyses on the structure, morphology and antigenicity of these multimers were carried out at different pH values during cell disruption and purification by size-exclusion chromatography. Process steps and environmental stresses in which aggregation or conformational instability can be detected were included. These analyses revealed higher stability and recoveries of properly assembled high-purity capsids at acidic and neutral pH in phosphate buffer. The use of stabilizers during long-term storage in solution showed that sucrose, sorbitol, trehalose and glycerol acted as useful aggregation-reducing agents. The VLP emulsified in an oil-based adjuvant were subjected to accelerated thermal stress treatments. None to slight variations were detected in the stability of formulations and in the structure of recovered capsids. A comprehensive analysis on scale-up strategies was accomplished and a nine steps large-scale production process was established. VLP produced after chromatographic separation protected rabbits against a lethal challenge. The minimum protective dose was identified. Stabilized particles were ultimately assayed as carriers of a foreign viral epitope from another pathogen affecting a larger animal species. For that purpose, a linear protective B-cell epitope from Classical Swine Fever Virus (CSFV) E2 envelope protein was chemically coupled to RHDV VLP. Conjugates were able to present the E2 peptide fragment for immune recognition and significantly enhanced the peptide-specific antibody response in vaccinated pigs. Overall these results allowed establishing improved conditions regarding conformational stability and recovery of these multimers for their production at large-scale and potential use on different animal species or humans.

## Introduction

Rabbit hemorrhagic disease (RHD) has for many years been responsible for the death or the slaughtering of free-living and domestic rabbits in different regions of the world [1]. The disease has become endemic in several countries and has also spread outside the original regions of appearance [2]–[6]. RHD provokes high mortality rates and a high number of pathologies in adult rabbits [7], [8]. The etiological agent is the rabbit hemorrhagic disease virus (RHDV), a non-enveloped and icosahedral calicivirus with a capsid mainly composed of the structural protein VP1 (VP60), of approximately 60 kDa [9].

During almost two decades, several approaches have been conducted to express the RHDV capsid protein in heterologous hosts or viral vectors aiming to reach a definitive non-conventional vaccine [10]–[24] that could also end animal welfare issues related with virus propagation in rabbits for vaccine preparation. However, only some of the systems assayed have genuine potential for the production and eventual licensing of a veterinary vaccine, a fact that depends on a great number of factors that include safety, technological, financial and policy issues.

Following the first outbreaks of RHDV in the Americas, our group directed research capabilities toward the development of a scalable production platform in order to ease the field introduction of a recombinant vaccine against RHD. Preceded by successful examples of subunit vaccine production and application in human and veterinary massive vaccination programs [25]–[27] one *Pichia pastoris* mutant strain was selected for the expression at high levels of the capsid protein from RHDV [22], [28]. The antigen was obtained forming soluble virus-like particles (VLP) inside the yeast cells with high antigenic resemblance to RHDV and attractive levels for a simple and inexpensive production process. At present, VLP vaccines against pathogens such as the human papillomavirus have been licensed and are commercially available [29].

The production of VLP for vaccination purposes should take into account some requirements related with structural integrity as stabilization of conformational epitopes and preservation of the multimeric state during large-scale purification, formulation or storage [30]. Bearing in mind these remarks we evaluated the structure of these VLP and characterized them during these processes under different conditions. Measurements of recoveries were carried out after each step and after stresses that had been shown to induce protein aggregation or conformational instability, thus allowing the establishment of optimal process conditions. The vaccine emulsions were also studied for their physical properties and antigen integrity after thermal stresses. A production process with high recovery levels is here proposed, developed, and validated through the evaluation of protective capacity. Potential stabilizers for these VLP were identified.

Recent investigations have also shown the significance of RHDV VLP obtained from cultured cells for the incorporation of foreign T-cells epitopes by genetic fusion or chemical conjugation and the generation of humoral and cell-mediated specific responses [31]–[34]. Therefore, an additional experiment was accomplished to investigate their feasibility as carriers of a B-cell epitope from Classical Swine Fever Virus (CSFV), an important disease in swine responsible for a highly contagious and fatal multisystemic disease that negatively impacts both animal health and economic issues worldwide [35], [36].

In our view, the pharmaceutically relevant improvements attained regarding structural stability, along with the establishment of a simple and inexpensive process for the large-scale production of RHDV VLP antigenically similar to RHDV provide researchers and manufacturers with valuable data for the development of carrier multimers for vaccination or therapeutic purposes. The multimers produced in this robust vaccine manufacturing platform have also the potential to become the first licensed yeast-derived VLP veterinary vaccine.

## Materials and Methods

### Yeast and Virus Strains

*Pichia pastoris* PVP12 strain [22] allowed the production of the recombinant VP1 multimers. VP1 obtained associated to the yeast membranous system of *P. pastoris* PVP11 strain [28] was used for comparative purposes in Western blot analyses. The RHDV Italian Reference strain Bs.89 (X87607) was used in competition enzyme linked immunosorbent assays (cELISA) while RHDV CUB5-04 strain (DQ841708) or the Spanish AST/89 isolate (Z49271) were employed in hemagglutination inhibition experiments. The latter strain was also used for the challenge of rabbits.

### Yeast Growth and Cell Disruption Procedures

The culture medium and growing conditions in the bioreactor were set essentially as previously described to induce VP1 production [28]. For disruption, the pellet was resuspended at 350 g L^−1^ (wet weight) in 50 mM phosphate buffer. A range of pH and different concentrations of NaCl were assayed. The disruption process was basically as described [28]. The raw disruption supernatant was clarified by a centrifugation step at 10 000 g, 4°C, and filtration through 0.2 µm.

### Purification of RHDV VLP by Size-exclusion High Performance Liquid Chromatography

For analytical purposes a size exclusion high performance liquid chromatography (sec-HPLC) TSK G5000 PW column (600 mm×7.5 mm) was used, equilibrated with phosphate buffer at a flow rate of 0.6 ml min^−1^. For purification of VLP at preparative scale a TSK G3000 PW column (1000 mm×67 mm) and a flow rate of 0.4 ml min^−1^ were employed. Detection was set at 280 nm and most profiles were acquired and processed using the LaChrom D-7000 HPLC System Manager v.3.1. The analysis of particle integrity purified under different buffer conditions or subjected to physical/thermal stressing treatments was also carried out by sec-HPLC. Prior to analytical experiments, VP1 was quantified in order to set an injection volume of 250 µL and sample concentration of 0.25 mg/mL.

### Production and Characterization of RHDV VLP

#### Purification of RHDV VLP on Sepharose CL4B

For scale-up or purification of larger quantities of RHDV VLP, a XK26/35 column (350 mm×26 mm) packed with Sepharose CL4B (Sigma, USA) was used, equilibrated with phosphate buffer at the appropriate pH. For production of active raw material starting from 10-L bioreactions a 1000 mm×300 mm column was used at flow rates of 5 mL/min.

#### Separation of RHDV VLP by Ultrafiltration

For comparative purposes, recombinant VLP were separated in a parallel experiment by tangential ultrafiltration using a Sartocon® Slice 200 ultrafiltration system having a Hydrosart membrane with a molecular weight cut-off of 300 kDa (Sartorius, Germany).

#### Influence of pH on the conformational stability and recovery of VLP during cell disruption, purification and post-purification steps

We tested a broad range of pH (3.0, 4.0, 5.0, 6.0, 7.0, and 8.0) during cell disruption and purification for their influence in aggregation, conformational distortion and loss in VLP final recovery. After cell disruption and size exclusion chromatography of the supernatant, VLP were subjected to a 10-fold concentration step using 300 KDa cut off membranes combined with diafiltration (changing three times the phosphate buffer volume) in a Sartocon® Slice 200 ultrafiltration apparatus, followed by centrifugation at 10000 g, 0.2 µm filtration, and quantification. Alternatively, three rounds of freezing/thawing were conducted after filtration. In all steps the recovery was determined. The experiments were performed in duplicate. Once the conditions regarding pH were evaluated an analysis on ionic strength and its influence on VLP recovery was performed. Different concentrations of NaCl (0.1, 0.3, 0.5, 0.7, 0.9 and 1 M) in the purification buffer were tested.

#### Screening of stabilizers for inhibition of protein aggregation and instability during storage

Various potential stabilizers generally recognized as safe (GRAS) were screened for their ability to prevent VLP aggregation and loss of protein from the final solution. To evaluate the efficacy of the excipients used, measurements of recovery were performed after purification, freezing/thawing, sterile filtration and storage at 4°C for a time period of twenty four weeks. A control lot containing VLP in phosphate buffer 0.3 M NaCl, pH 7.0 without excipients was used for comparison. The additives used were: tween 20 (0.05 and 0.01%) sodium citrate (0.1, 0.2 M), and sucrose, trehalose, sorbitol and glycerol (at 10 and 20%). The experiments were conducted using duplicate lots in each case.

#### Equilibrium density gradient centrifugation

The study of particles integrity in equilibrium sucrose density gradients was conducted in duplicate as previously described [22].

#### Detection of VLP epitopes

The antigenic characterization of the VLP was conducted by sandwich ELISA using a panel of horseradish-peroxidase conjugated monoclonal antibodies directed against RHDV conformational epitopes, supplied by the OIE (Office Internationale des Epizootes) reference laboratory for RHD (Brescia, Italy). The characteristics of the mAbs are as follows: mAb 1H8 recognizes an external, protective epitope present only in correctly assembled RHDV, and absent in aberrant or disassembled capsids. MAb 6F9 recognizes another protective epitope in RHDV. MAbs 6H6 and 5B2 recognize conformational surface epitopes present in monomeric VP1. MAb 6G2 is directed against a buried epitope located at VP1 N-terminal portion. The mAbs working dilution was 1:500. In sandwich ELISA, hyperimmune serum diluted 1:500 against RHDV AST/89 was used for coating the plates. Identical quantities of VP1 (one microgram per well) were loaded for the analysis of surface epitopes. The assays were performed in duplicate for the different samples analyzed. If needed, the presence of heterogeneous VP1 populations was verified employing mAb 6H6.

#### Quantification

The expression levels of VP1 were measured by sandwich ELISA using anti-RHDV hyperimmune serum at a dilution of 1:3500 as capture antibody, the monoclonal antibody 6H6 (dilution 1:500) for antigen detection, and a curve of different known concentrations of standard VP1 from insect cultured cells [23]. For quantification of assembled VLP the monoclonal antibody 1H8-HRP conjugate was used. Reactions were developed for 3–5 min in the dark using 0.4 mg mL^−1^ orthophenylenediamine (OPD) (Sigma, St. Louis, USA) diluted in 0.005 M citric acid/0.1 M Na_2_HPO_4_ containing 0.015% H_2_O_2_ (BDH, UK). The reaction was stopped with 50 µL per well of 2.5 M H_2_SO_4_. Measurements were performed at 492 nm in a SensIdent Scan ELISA reader (Merck, Germany).

### Vaccine Formulation

VLP were quantified and the final solution was sterilized by filtration. The oil-based adjuvant Montanide 888 VG (SEPPIC, France) was mixed in sterile mineral oil at a ratio of 1:9. A water-in oil emulsion was prepared at a proportion of 40% oil phase and 60% aqueous phase using the homogenizer ULTRA-TURRAX T25 basic (IKA Works Inc., NC). Depending on the experiment accomplished, vaccines were formulated as 5, 10, 25 and 50 µg in a final volume of 1 mL per dose. They were filled manually under sterile conditions in glass bottles of 20 mL.

### Mechanical Stability

To determine the stability of the emulsion by gravitational force effects, 10 mL of emulsion were poured into two 15 mL centrifuge tubes. The height of the emulsified column (*Ho*) was measured in the tubes and then they were centrifuged in a 30 cm radius centrifuge for 1 hour at 3000 rpm. The emulsified column height was measured again (*Hu*). The ratio *Hu/Ho* was calculated. Only if this relation was equal to or higher than 0.8 the sample was considered to fulfill quality parameters of production.

### Thermal Stress Experiment

Samples of vaccine formulations were incubated for 7 days at 4°C, 37°C, 48°C or 60°C, until further studies.

#### Thermal stability

The height of the emulsified column (*Ho*) was measured prior to the stresses. Then, only the emulsified column was measured again (*Hu*). The *Hu/Ho* ratio was calculated. Only if the relation was equal to or higher than 0.9 it was considered that the sample fulfilled the quality parameters.

#### Viscosity and organoleptic characteristics determination

Viscosity measurements for the formulated samples subjected to thermal stress were recorded at days 0 and 7 using a rotational rheometer. A cp 40 spindle was used and the gradient speed employed was of 2, 4, 6, 8 and 10 rpm. A visual evaluation of organoleptical characteristics from each sample was carried out. The study was performed at 25°C.

#### Emulsion separation

VLP/Montanide 888 VG emulsion samples of 1 mL each were frozen at −20°C for 12 h. After centrifugation at 10 000 g for 30 min the samples were separated in two phases; an aqueous phase (translucent) and a semi-oil phase (white). The aqueous phase was extracted and stored at −20°C. For the analysis of the VLP surface epitopes within the aqueous phase, one microliter was used in ELISA.

### Transmission Electron Microscopy

The morphology and size of RHDV VLP in samples purified at different pH values or subjected to thermal stresses was examined by transmission electron microscopy (JEOL JEM 2000 EX, Japan) as already described [22].

### CSFV Peptide Coupling to RHDV VLP

#### Peptide synthesis

The 24^th^ amino acids CSFV E2 peptide (H_2_N-KEDYRYAISSTNEIGLLGAEGLTC-COOH) was synthesized at analytical scale using the Fmoc/tBu strategy [37] at the Peptide Synthesis Laboratory of CIGB. It was purified by RP-HPLC and characterized by mass spectrometry. The mass spectrum was acquired in a hybrid Q-Tof -2™ mass spectrometer from Micromass (UK) equipped with an ESI Z-spray ion source. The software for acquisition and processing of the mass spectra was MassLynx, v4.0 (Waters, USA). Peptide RP-HPLC purity grade was of 95% and its identity was confirmed by comparison with the theoretical value for this amino acids sequence.

#### Coupling to the carrier protein

A Cysteine amino acid coupled at the C-terminal of the peptide sequence was used as a convenient handle for peptide conjugation to the carrier protein. Briefly, VP1 was dissolved in phosphate buffer solution pH 6.0 and a 10 mg/mL solution of M-maleimidopropionyl-N-hidroxysuccinimide esther (MPS) in N,N-Dimethylformamide (DMF), was added. The reaction was stirred during 30 min at room temperature. Free MPS was separated from MPS-activated protein by dialysis against phosphate buffer solution pH 6.0 at 4°C. Then, CSFV E2 peptide dissolved in phosphate buffer solution pH 6.0 was added to the previously activated protein and the reaction was stirred for 3 hours at room temperature. Free CSFV E2 peptide was separated from the peptide conjugated protein by dialysis against PBS (pH 7.2) at 4°C. Both dialysis steps were performed with a Spectra/Por® molecular porous membrane (MWCO 12–14 000) from Spectrum Laboratories, Inc. (USA & Canada). Total protein content was determined by the Lowry method [38]. Characterization of CSFV E2 peptide attachment to VP1 was carried out by analysis in SDS-PAGE and sec-HPLC.

### Immunization Trials

#### Animals

New Zealand female rabbits of 8 weeks age and approximately 2 Kg weight, seronegative to RHDV (as determined by ELISA) were used in all the experiments. Eight to 10 weeks old Duroc/Yorkshire crossbreed male pigs, serologically negative to CSFV and belonging to a non-vaccinated and CSF free herd were also used. All the studies involving experimentation with animals were in accordance with guidelines and recommendations from the Guide for the Care and Use of Laboratory Animals (current Edition) and policies from the Cuban Society of Laboratory Animals’ Science (SCCAL). The experimental protocols were drafted by the authors and approved by the Animal Welfare Commission of the Bioterio Division from the Center for Genetic Engineering and Biotechnology. In all cases, supervision of veterinary authorities from the Institute for Veterinary Medicine, Havana, Cuba was guaranteed. When rabbits or pigs were used for experimentation, they were housed in individual rooms and appropriate feeding, water supply and health monitoring was permanently provided. Animals euthanized were humanly handled. They were subjected to initial anesthetization and potassium chloride injection intravenously to avoid suffering after challenge. Pigs were also carefully monitored for body temperature and any signs of immunization reactions.

#### Serum samples procedure

Blood samples were extracted from the marginal ear vein of rabbits at days 0, 14, 21, 40, and also at 60, 90 depending on each trial, in order to analyze the titers, specificity and time-course of antibodies generated against RHDV. In trial II, serum samples were taken to survivors 10 days after challenge.

#### Experiment I: Immunogenicity of the formulation subjected to thermal stress

Six groups of six rabbits each were immunized with vaccine formulations treated at 4°C, 37°C, 48°C or 60°C for one week, respectively. The animals received two subcutaneous doses containing 50 µg of the protein at days 0 and 21. The experiment included a negative control group that was simultaneously injected with Montanide 888 VG as placebo and a group immunized with the vaccine formulated at the time of use.

#### Experiment II: Protective efficacy

Three groups comprising one group of seven rabbits and two groups of three rabbits each were immunized as follows: Group I (seven rabbits) was immunized with two doses containing 50 µg of the VLP. The second group was vaccinated with 50 µg of VP1 expressed in *Sf9* cultured cells using the baculovirus expression system, and a negative control group was injected with Montanide 888 VG. Rabbits were injected via subcutaneous at days 0 and 21. At day 40 they were intramuscularly challenged with a lethal dose of approximately 100 LD_50_ (16 000 HA units) of the AST/89 RHDV strain. Challenged animals were monitored for clinical symptoms of the disease.

#### Experiment III: Minimal protective dose

An experiment was designed to investigate the minimum dose and the number of vaccine inoculations needed to achieve the protective response. Seven groups of six rabbits each were subcutaneously immunized with vaccines formulated to contain different quantities of VLP: From groups 1 to 4, rabbits were injected with two doses separated by 21 days. Group 1 received 50 µg, group 2 was immunized with 25 µg, group 3 was injected with 10 µg and group 4 received doses of 5 µg. Groups 5 and 6 were immunized with a single dose containing 50 and 25 µg, respectively while a sixth group was solely injected with Montanide 888 VG as placebo.

#### Experiment IV: Immunization with RHDV VLP-E2 peptide conjugate

This experiment was identically carried out in rabbits and pigs. Five groups of five animals each were randomly generated and immunized as follows: All vaccine preparations were emulsified in Montanide 888 VG in a final volume of 1 mL; all animals were immunized by intramuscular injection in the neck (pigs) or the back (rabbits) and doses were administered two weeks apart. Group 1 received 50 µg of unconjugated RHDV VLP as placebo. Group 2 was immunized with 50 µg of the entire E2 recombinant protein purified from the milk of non-transgenic goats [39]. Group 3 was injected with 50 µg of monomeric E2 peptide. Group 4 was vaccinated with 50 µg of unconjugated VLP co-formulated with 50 µg of the soluble E2 peptide. Group 5 was vaccinated with 50 µg of RHDV VLP carrying the E2 peptide fragment attached by chemical conjugation. Sites of injection and animal health status were monitored daily.

### RHDV Antibody Assays

#### Competition enzyme-linked immunosorbent assay

Sera from rabbits were evaluated at a single dilution (1:10) with a competition ELISA for RHD [40] manufactured at the OIE-Reference Lab, Italy, implemented as previously described [22]. Sera were considered negative when the absorbance value at 492 nm decreased by less than 15% with respect to the reference value (average of negative control sera) and positive if they decreased by 25% or more (OIE Terrestrial Manual, 2010).

#### Hemagglutination inhibition assay

Serum hemagglutination inhibition (HI) titers were measured as already described [22]. Positive controls (sera from rabbits immunized with inactivated RHDV CUB5-04) and negative controls (sera collected from seronegative rabbits) were included. The HI titer of each sample was expressed as the reciprocal of the highest dilution at which no hemagglutination was observed.

### Detection of Peptide and E2 Specific IgG Antibodies by ELISA

In brief, to determine antibody levels against E2 whole protein or E2 peptide in rabbits or pigs serum, PolySorp F96 immunoplates (Nunc, Denmark) were coated for 12 h at 4°C with 100 µL per well containing 1 µg of the appropriate antigen in 0.1 M carbonate buffer, pH 9.6. Plates were washed and blocked with 2% skim milk (Oxoid, UK) for 1 hour. One hundred µL of serum from each animal was added at a dilution 1:10 in PBS and serially diluted two fold in replicate wells for titration. Plates were incubated, washed, and 100 µL of goat anti-rabbit or rabbit anti-swine IgG-horseradish peroxidase conjugates (Sigma-Aldrich, USA) diluted 1:10000 in PBS were added to each well. Reactions were developed with OPD and read at 492 nm. Titers were expressed as the inverse of the maximum serum dilution having an OD superior to the negative control mean OD. A modified cELISA was adapted to detect anti-RHDV VLP IgG antibodies in serum of immunized pigs by addition of swine serum for competitive analysis.

### Statistical Analysis

Mean antibody titers, mean absorbance values or mean inhibition values were obtained from serum of individual animals, according to each experiment described. If applicable, an analysis of variance was employed for each time point and mean values were compared using the Newman-Keuls Multiple Comparison test. Otherwise, values were compared with the Kruskal–Wallis and the Dunn’s tests. The tests were carried out using the statistical software GraphPad Prism v4.0.

## Results

### Variations to pH, Ionic Strength and the Use of Additives Influence the Stability and Recovery of RHDV VLP during Cell Disruption, Purification and Storage

The structure of multimers antigenically similar to RHDV obtained in *P. pastoris* was analyzed at different pH values during cell disruption and purification at a temperature of 25°C. Quantifications of the VLP were conducted at various stages considering that some stressing steps were previously shown to induce aggregation phenomena or conformational instability. Structure preservation of the VLP during storage in solution was also examined with the use of stabilizers with potential value in a large-scale production process.

Through the evaluation of a range of pH from 3 to 8 different chromatographic profiles were recorded by sec-HPLC using a TSK G3000 PW preparative column. In the process assayed, clarified disruption supernatant from yeast cells was taken as starting material. The VLP were first quantified followed by a single chromatography step, concentration/diafiltration, sterile filtration, re-quantification and conformational analysis. Otherwise, three rounds of freezing/thawing were included in the process followed by sterile filtration and antigenic studies. Measurements of the final recovery were conducted.

**Figure 1:**
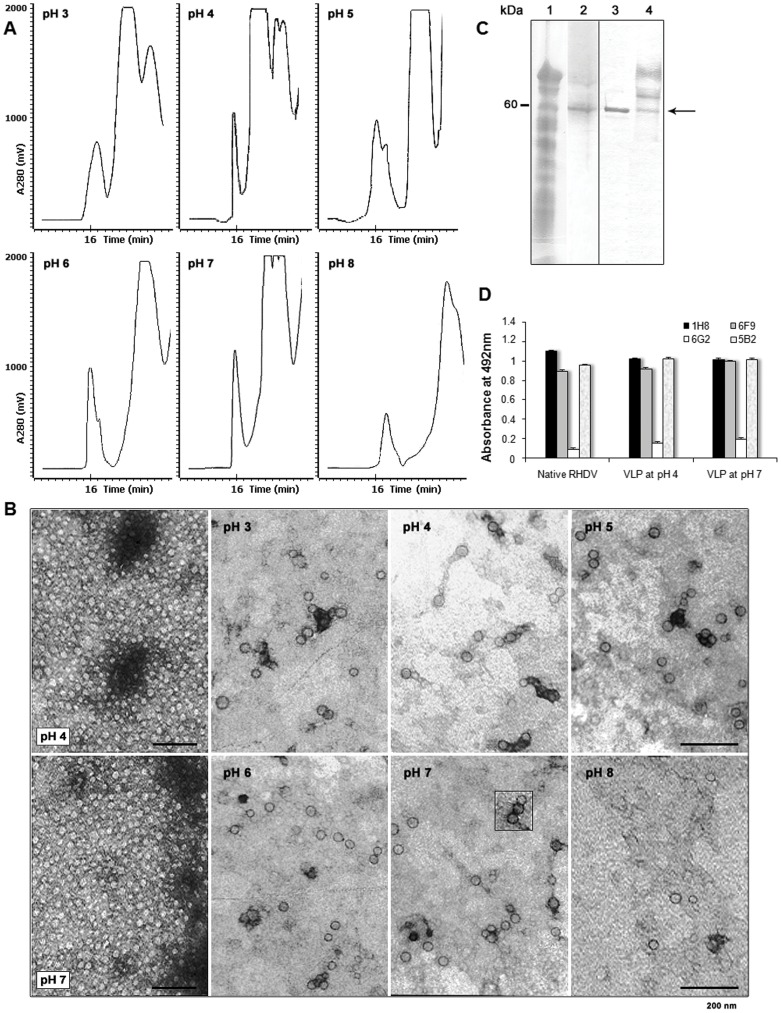
Analysis of the RHDV VLP purified under different conditions. (**A**) Chromatographic profile of RHDV VLP after disruption and purification conducted at different pH values. Purification was performed by sec-HPLC using a TSK G-3000 preparative column. In all cases multimeric VP1 was detected in the first peak and quantified by sandwich ELISA using monoclonal antibodies. Retention times are indicated. (**B**) Electron micrographs of negatively stained samples containing VP1 multimers obtained after separation in sec-HPLC. According to measurements by ELISA, the highest recoveries were obtained at pH 4 and 7. The morphology and size of the VLP was analyzed by TEM using 2% uranyl acetate for negative stain. The bars indicate 200 nm. Magnifications: x30 000 and x40 000. (**C**) SDS-PAGE (lanes 1, 2) and Western blot analysis (lanes 3, 4) of purified VLP. Lane 1 corresponds to disruption supernatant from *P. pastoris* PVP12 strain. Lanes 2 and 3 were loaded with purified RHDV VLP. A minor band migrating close to 60 kDa was also detected in lane 3. For comparison, lane 4 shows glycosylated VP1 expressed associated to the yeast membranous system in PVP11 strain, in which various protein bands were recognized by the hyperimmune serum. The molecular weight of approximately 60 kDa is shown in the left and indicated by an arrow in the right. (**D**) Analysis with conformation dependent monoclonal antibodies of epitopes located on the VLP obtained after cell disruption, purification, and rounds of stressing steps at pH values of 4 and 7. RHDV VLP and native RHDV were detected using mAbs 1H8, 6F9, 5B2, and 6G2. Standard deviation bars of measurements performed are indicated.

The highest quantities of multimers measured after purification and concentration were found at pH 4 and 7 with recoveries of about 84 and 88%, respectively. One homogeneous, undistorted narrow peak corresponding with RHDV VLP at a retention time of approximately 16 min was seen in both cases, as detected with mAb 1H8. On the opposite, the chromatography pattern varied the most at pH 8 showing a minor peak and fewer particle yields. High to moderate recovery levels were obtained at pH 6, 3 and 5, with a decrease in that order (**Table 1**). Some variations with respect to the profiles obtained at pH 4 and 7, consisting on heterogeneity due to the presence of more than one population of VP1 were also seen, mostly at pH 5 (**Figure 1A**). The decrease in recoveries of the multimers at some pH values was attributed to the formation of insoluble aggregates. In the purifications carried out, after concentrating the volumes recovered from the chromatography column and once more after freezing and thawing, small, insoluble white flocks could be visualized and separated by centrifugation or filtration. The presence of VP1 in those fractions was corroborated by ELISA and immunodot. Insoluble VP1 without its proper conformation accounted for about two thirds of the protein quantified while VP1 with native unaffected epitopes accounted for approximately one third. These solutions, which became more opalescent to the eye, produced a substantial light absorption increase in turbidity measurements conducted at 380 nm (not shown). Aggregation was also induced and confirmed in turbidity measurements carried out at 60°C. Finally, after freezing/thawing stresses, the processes at pH 4 and 7 rendered the highest recoveries of assembled capsids, with values over 40 and 45%, respectively **(** **Table 1**).

**Table 1:** Recovery of properly assembled RHDV VLP during the evaluation of different pH in the disruption and purification processes.

VLP quantification [Mean ± standard deviation (µg/mL, first row)/Percent of recovery (%, second, third row)]						
Process stage	pH 3.0	pH 4.0	pH 5.0	pH 6.0	pH 7.0	pH 8.0
Cell disruption, centrifugation, and filtration (0.2 µm)	452±19.6	850±35.3	415±18.3	685±25.1	820±31.2	318±12.0
Size exclusion chromatography, 10 fold concentration-diafiltration, centrifugation, sterile filtration (0.2 µm)	57.5±6.4	84.2±5.6	43.2±4.8	68.5±7.0	88.5±5.1	44.8±4.1
Three rounds of freezing-thawing, sterile filtration (0.2 µm)	33±4.2	40.6±5.6	29.7±8.1	35.7±6.4	45.4±2.6	24±3.5

Purification was performed by size exclusion chromatography using a preparative TSK G3000 column. Alternatively, an additional step known to induce instability and protein aggregation (rounds of freezing-thawing) was included. Quantification of recovered VLP was accomplished by sandwich ELISA with the use of mAbs.

VLP directly detected after cell disruption and clarification were expressed in the first raw as µg per mL of disruption supernatant. Starting from those values, percents of recovery are shown for the next two steps. All measurements were done in duplicate starting from two independent bioreactions. Protein amounts and percents of recovery refer only to properly assembled VLP.

To further characterize changes induced by pH, we examined the morphology of the multimers, as well as the preservation of surface epitopes which are relevant for the proper tertiary and quaternary state of the protein. After transmission electron microscopy (TEM), the multimeric structures seemed predominantly invariant in the pH range 3–7 showing an structure with diameters of around 30–40 nm in most cases (**Figure 1B**). At pH 8, fewer particles were distinguished, suggesting that the alkaline pH started to induce changes in VP1 quaternary structure. The latter results were consistent with buoyant densities recorded, in which values closer to the buoyant density of authentic VLP were achieved at pH from 3 to 8, with the closest values (1.31–1.32 g/mL) at pH 4–7. Conformational epitopes were also detected by monoclonal antibodies 1H8, 6F9, 6G2 and 5B2 with a variety of recognition degrees that were in accordance with the results obtained by means of the other techniques. The RHDV VLP produced at pH 7 were also characterized by SDS-PAGE and Western blot analysis (**Figure 1C**). The multimers obtained at pH 4 and 7, as well as native virions (Bs.89 strain) used as positive control were identically recognized by the mAbs above mentioned (**Figure 1D**).

Variations to the ionic strength of the buffer species selected (sodium phosphate) and their influence on VLP stability suggested that a concentration of 0.3 M NaCl is the most appropriate in terms of final recovery. This analysis was conducted at pH values of 4 and 7 (**Table 2**). After that, a number of excipients were evaluated at different concentrations for their ability to prevent aggregation or destabilization of the VLP structure during a 24 week storage period at 4°C (pH 4 and 7). Under such conditions, a similar event of protein loss had been observed. Although the margin of variation was to some extent narrow, the results indicated that sucrose 20%, sorbitol 20%, glycerol 20%, trehalose 20% and 0.2 M sodium citrate were at different degrees useful stabilizing agents in comparison with the use of no additives during long-term storage in water buffered solution. According to the experiment, when no additives were used a recovery of approximately 50% is encountered. In summary, phosphate buffer containing 0.3 M NaCl at either pH 4 or 7 can be used to obtain major recoveries of multimeric VP1.

**Table 2:** Recovery of VLP obtained after buffer variations and with the use of stabilizers during storage.

Treatment	VLP percent of recovery (%)					
Purification in phosphate buffer, including three rounds of freezing/thawing	0.1 M NaCl	0.3 M NaCl	0.5 M NaCl	0.7 M NaCl	0.9 M NaCl	1 M NaCl
At pH 4.0	35.7	44.8	42	40.4	42.9	43.2
At pH 7.0	23.4	46.5	32.7	34.1	29.8	31
**Additives in the final storage volume (at 4°C, pH 7.0 for 24 weeks)** a	**Tween 20**	**Sodium citrate**	**Sucrose**	**Trehalose**	**Sorbitol**	**Glycerol**
0.05%	24.5					
0.01%	10.5					
0.2 M		56.2				
0.1 M		28.0				
20%			71.9	69.4	70.1	75.4
10%			48.4	49.0	47.5	55.6

a Various excipients were evaluated at different concentrations during VLP in storage at 4°C for a time period of 24 weeks after purification. The initial concentration of VLP in all samples was 500 µg mL^−1^. The experiments were conducted using duplicated lots. The percent of recovery was calculated as the percent of aggregation inhibition in the presence of additive = aggregation inhibition in the presence of additive * 100/concentration of aggregated proteins in the absence of additive where:

aggregation inhibition in the presence of additive = concentration of aggregated proteins in the absence of additive - concentration of aggregated proteins in the presence of additive.

Similar results in terms of recovery were obtained at pH 4 or 7 with the additives assayed. For simplicity, only recoveries at pH 7.0 are shown.

As a control lot, VLP were kept in identical storage conditions without stabilizers in phosphate buffer pH 7.0, 300 mM NaCl. In such case, the recovery of VLP accounted for a 48.5%.

### The Vaccine Formulation Containing the Recombinant VLP Remained Physically and Thermally Stable after an Accelerated Thermal Stress Experiment

RHDV VLP were formulated in Montanide 888 VG. The emulsion obtained after mixing complied with the Oswald model for non-Newtonian fluids, with viscosity-index values (µ) <1500 mPa/s and flow-index values (n) between 0.5 and 0.9. The formulated vaccines were subjected to an accelerated thermal stress treatment and then studied for the physical properties of the emulsion and integrity of the multimers.

**Figure 2:**
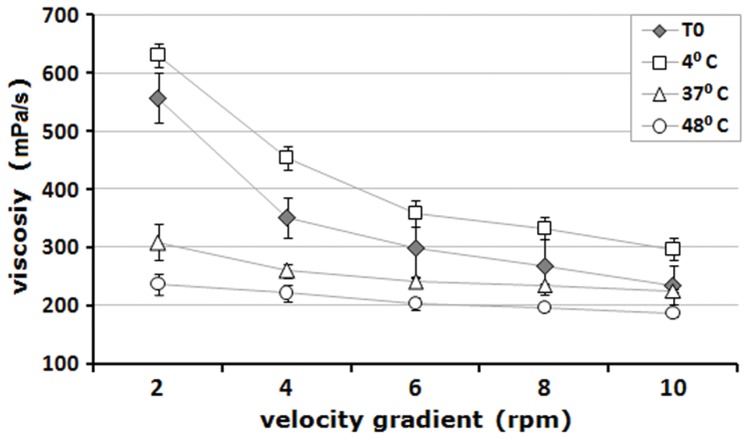
Evaluation of the apparent viscosity in a velocity gradient of RHDV VLP vaccine samples treated at 4°C, 37°C, and 48°C for 7 days. An emulsion sample taken immediately after formulation was used as control. The average and standard deviation bars for each experimental point are indicated. T0 =  time 0.

The physical stability of the formulations was first assayed through viscosity analyses. In those emulsions kept at 37°C or 48°C for 7 days, this parameter was significantly lower than that of the emulsion maintained at 4°C. The emulsion stored at 4°C had a similar, although a slightly higher viscosity than the vaccine emulsified and evaluated immediately after formulation, which was used as a control (**Figure** **2** **)**. On the other hand, the emulsion incubated at 60°C became separated into two phases. No differences were observed in the organoleptical characteristics of the unseparated emulsions, which at the end of the experiment showed a non-Newtonian fluid behaviour, as oil-based formulations do.

**Figure 3:**
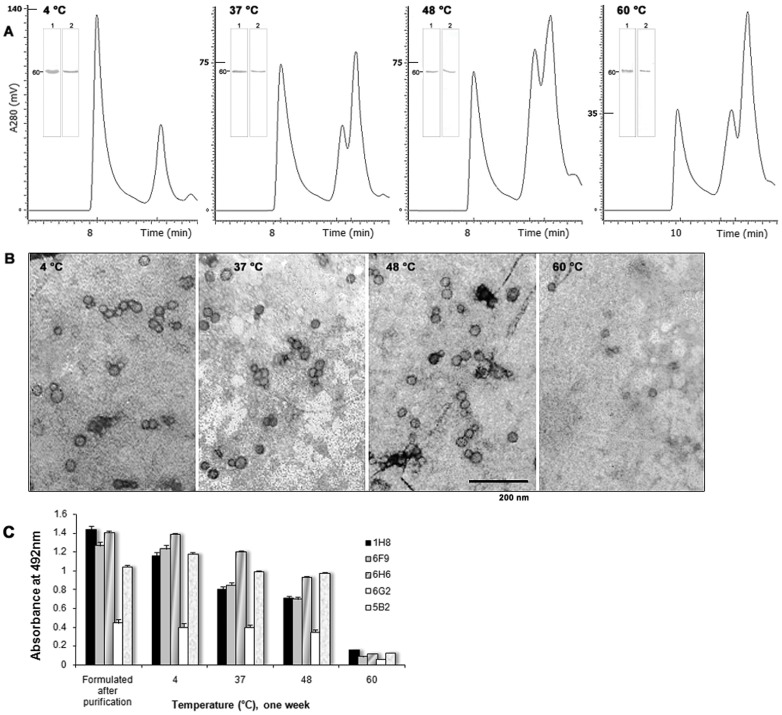
Analysis of the recombinant VLP extracted from vaccine formulations subjected to an accelerated stress treatment experiment. (**A**) Analysis in size-exclusion HPLC. The temperature at which the emulsion was kept for one week and the retention times corresponding to elution of multimeric VP1 are indicated in each chromatogram. The inner figures show Western blot analyses and detection of VP1 in the main peaks detected, which were designated as 1 and 2 according to their appearance in the chromatogram. An anti-RHDV hyperimmune serum was used. The 60 kDa band is indicated. (**B**) Electron micrographs of negatively stained samples containing the extracted VLP. The VLP structure was analyzed by TEM using 2% uranyl acetate. The bar indicates 200 nm. Magnification: x40 000. (**C**) Detection of RHDV VLP epitopes with the conformational-sensitive monoclonal antibodies 1H8, 6F9, 6H6, 5B2, and 6G2 after thermal stresses. Standard deviation bars are indicated.

VLP were extracted from the emulsions treated and analyzed by sec-HPLC. In the samples treated, a reduction in the intensity of the signal corresponding to VLP was noticed although retention times remained invariable (**Figure 3A** **)**. An increment in the homogeneity of the high molecular weight structures from samples kept at 48 and 60°C was observed while an increment in late signals, attributed to dissociated capsids and not to degradation, was detected for samples subjected to these temperatures.

The appearance and structure of the multimers was analyzed by TEM and no evident changes were seen in samples incubated at 4, 37 or 48°C. In samples kept at 60°C, small quantities of assembled high molecular weight structures were observed (**Figure 3B**). Results from density gradient centrifugation experiments of samples treated at 37 and 48°C revealed a slight decline of VLP concentration in those fractions with buoyant densities close to that of RHDV. This decline became clear at 60°C. Changes in the quaternary structure of VLP stressed at this last temperature were apparently irreversible. However, the study on the preservation of conformational epitopes revealed minor variations in the reactivity of mAbs 1H8, 6F9, 6H6, 5B2 and 6G2 in samples incubated at 37 or 48°C. As expected, the epitopes recognized by these mAbs were hardly detected in the sample kept at 60°C (**Figure 3C**). These experiments demonstrated that differences can be found; nonetheless the vaccine formulations remain stable at temperatures of 37 and 48°C. As later evidenced in animal experiments, the slight variations in the conformational structure of the VLP within these formulations were not significant in terms of immunogenicity.

### Size-exclusion Chromatography on Sepharose CL4B is an Efficient Approach for the Large-scale Production of RHDV VLP

Recombinant RHDV VLP could be purified and processed in accordance with the previous findings in order to obtain the highest recovery and stability levels. Laboratory-scale procedures and analytical techniques were selected and summarized in view of the process scale-up. Then, 10-L culture bioreactions were carried out as two independent lots. Depending on their characteristics, the subsequent operations were also scaled-up. Production of VP1 multimers was in accordance with the seed-batch principle, in which the recombinant strain is maintained as a master seed bank (MSB) and working cell bank (WCB) and are both stored in individual vials at −70°C. The stock is stored for many years and each new culture starts from the same original seed preparation. These cell banks comply with sterility and viability tests performed periodically, showing 10^6^ cells/mL in every determination.

**Figure 4:**
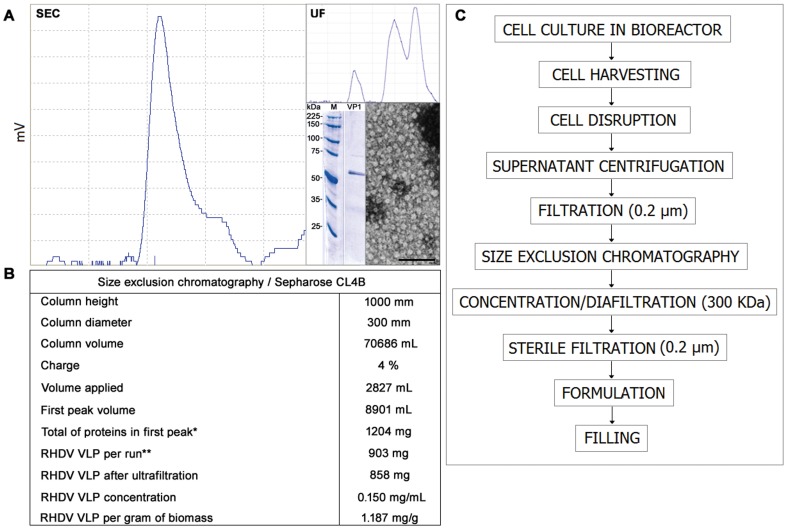
Purification scale-up of the recombinant RHDV VLP by size exclusion chromatography (SEC) on Sepharose CL4B, starting from 10-L culture bioreactions. (**A**) Chromatography pattern of homogeneous high-purity VLP. The inner upper figure corresponds to purification by tangential ultrafiltration (UF) using a membrane with a 300 kDa cut-off, showing low purity VLP. The inner lower figure shows SDS-PAGE and electron micrograph of negatively stained samples containing VLP purified by SEC. Numbers in the left indicate the migration of standard molecular weight markers. The bar indicates 200 nm. Magnification: x30 000. (**B**) Results obtained from the VLP production scale-up. (*) Total of proteins measured by the Lowry method. (**) Properly assembled RHDV VLP quantified by ELISA. (**C**) Block diagram showing upstream and downstream processes to obtain the recombinant VLP and to produce the vaccine at large-scale.

For purification scale-up, separation of the VLP was performed by gel filtration chromatography using Sepharose CL4B. Tangential ultrafiltration of clarified supernatant using the Sartocon® Slice 200 ultrafiltration system with a membrane with a molecular weight cut-off of 300 kDa was carried out in parallel for comparative purposes. Interestingly, purity of ordered multimers obtained in the single chromatography step was over seven times higher (72.04 versus 10.2) than purity achieved by ultrafiltration (**Figure 4A**). The total of proteins in the first peak was determined by the Lowry method [38], followed by quantification of RHDV VLP by ELISA using the monoclonal antibody 1H8 (**Figure 4B**). In this way, VP1 quantities are only referred to properly assembled capsids. In the first peak, a minor amount of heterogeneous VP1 detected with monoclonal antibodies other than 1H8 accounted for the rest of the protein purified while alcohol oxidase from *P. pastoris* appeared as a faint band migrating at approximately 75 kDa. The elution of lower molecular mass proteins and yeast components was obtained as a highly dense yellow solution in the last fractions. As suggested in the Test Procedures and Acceptance Criteria for New Biotechnological/Biological Veterinary Medicinal Products from the International Cooperation on International Harmonization of Technical Requirements for Registration of Veterinary Medicinal Products (VICH), heterogeneous VP1 was considered as a product-related substance. Then, VLP were finally separated in Sepharose CL4B following process hints and established manufacturing practices approved for the large-scale production of the licensed recombinant Gavac^plus^™ vaccine [41], [26]. The disruption and purification steps were carried out in one day and allowed producing active raw material equivalent to approximately 50000 doses per bioreaction. At the end, samples were tested for sterility and potency in mice and rabbits taking cELISA and HI assays as tests to comply (not shown). The process, developed until formulation and filling, was conducted with good reproducibility and set the bases for larger production. A flux diagram of the nine steps procedure was established (**Figure 4C** **)**. The aqueous part of the final formulation consisted of phosphate buffer 0.3 M NaCl, pH 7.0 containing the VLP, then adjuvanted in Montanide 888 VG. Vaccine preparations complied with mechanical and stability tests. Various trials were carried out in order to complement the results described.

### The Vaccine Formulations Assayed were Highly Immunogenic in Vaccinated Animals

The first trial aimed to investigate the influence of the thermal stresses applied to the VLP on their immunogenicity in rabbits. After treatments to the formulations, rabbits were immunized at days 0 and 21 with 50 µg of VLP. A cELISA was used to detect specific anti-RHDV IgG levels. Except for the group immunized with the vaccine treated at 60°C and re-formulated, all the animals vaccinated with emulsions prepared at the time of use, or kept at 4, 37, or 48°C for one week, developed antibody levels corresponding with inhibition percents over 85–90% in determinations practiced at days 14, 21, 40, 60, and 90. No statistically significant differences were found between them at each time point investigated.

Rabbits for the challenge experiment were immunized under the same schedule. Animals injected with insect cells-derived VP1 or placebo were included as positive and negative controls. All animals were challenged 42 days after the first vaccination with 100 LD_50_ of RHDV. Prior to challenge, all rabbits receiving the yeast-derived VLP and rabbits from the positive control group had developed anti-RHDV specific antibodies with inhibition percents over 90% and HI titers up to 1/1024. Titers slightly increased after challenge in animals immunized with the yeast-derived VLP up to values that ranged from 1/512 to 1/2048 (**Table 3**). Rabbits receiving placebo died within the first 36 hours after challenge or were euthanized to avoid suffering. Clinical symptoms of the disease and visible lesions on spleen and liver during necropsy were observed only in rabbits injected with placebo.

**Table 3:** Hemagglutination inhibition titers detected in sera of rabbits immunized with the recombinant VLP and challenged with RHDV.

Anti-RHDV hemagglutination inhibition titers^a^/Anti-RHDV ELISA inhibition percents [Mean ± standard deviation]^b^			
Days	Yeast-derived VLP vaccine (n = 7)	VP1 (VP60) from insect cultured cells (n = 3)	Placebo (n = 3)
0	n.d.	n.d.	n.d.
Mean	10.4±2.5^a^	8.7±4.6^a^	9.4±2.8^a^
40	1/256	1/4096	1/16
	1/1024	1/2048	1/16
	1/256	1/1024	1/8
	1/256		
	1/1024		
	1/512		
	1/128		
**Mean**	**91.5±1.6** a	**92.8±3.7** a	**10.1±4.6** b
10 days post-challenge	1/1024	1/8192	–
	1/2048	1/4096	
	1/512	1/4096	
	1/2048		
	1/2048		
	1/256		
	1/512		
**Mean**	**93.9±0.7** a	**94.2±1.4** a	**–**
Survival after 10^2^LD_50_ RHDV challenge	100%	100%	0

a The HI titer was expressed as the maximum dilution capable to completely inhibit the agglutination of human type O erythrocytes by RHDV. Each titer value corresponds to an individual animal. In the assay, a hyperimmune serum against RHDV (with an HI titer of 1/2048) was used as positive control. All animals were immunized at days 0 and 21. n.d.: hemagglutination inhibition titers not detected.

b The result is also expressed as the mean ± standard deviation of inhibition values obtained in cELISA from individual animals. Different letters within a row indicate statistical significant differences for *p*<0.05, according to the Kruskal-Wallis and Dunn’s tests.

A further experiment explored whether a reduction in the number of doses or in antigen quantities could elicit protective immunity. Groups of rabbits were immunized twice with 5, 10, 25 or 50 µg of VLP. Two other groups were vaccinated with a single VLP dose of 25 or 50 µg. HI assays and cELISA were used to measure the immune responses. Rabbits injected twice with 5 or 10 µg of VLP were able to develop an antibody response with inhibition percents over 85% in cELISA although protective HI titers over 1/80 were not detected. In contrast, animals immunized twice with 25 or 50 µg per dose showed inhibition percents over 85% and protective HI values up to 1/512. Interestingly, inhibition percents and HI titers elicited in rabbits inoculated once with 50 µg of the VLP were similar to those developed in animals immunized twice with the same dose. They were also in the range of values from animals injected twice with 25 µg, which in turn were similar to those detected in rabbits vaccinated once with that dose (**Table 4**).

**Table 4:** Hemagglutination inhibition titers detected in sera of rabbits vaccinated with different doses or different VLP concentrations per dose.

Anti-RHDV hemagglutination inhibition titers^a^/Anti-RHDV ELISA inhibition percents [Mean ± standard deviation]^b^							
Days	Two doses 50 µg	Two doses 25 µg	Two doses 10 µg	Two doses 5 µg	One dose 50 µg	One dose 25 µg	Placebo
0	n.d.	n.d	n.d	n.d.	n.d	n.d.	n.d.
Mean	7.2±2.5^a^	5.8±3.4^a^	11.4±4.1^a^	9.8±2.2^a^	6.9±5.7^a^	4.2±3.5^a^	10.3±4.2^a^
40	1/64	1/128	1/256	1/32	1/128	1/128	n.d.
	1/512	1/128	1/128	1/32	1/1024	1/128	
	1/128	1/64	1/16	1/32	1/128	1/64	
	1/128	1/128	1/32	1/64	1/128	1/128	
	1/512	1/128	1/32	1/8	1/128	1/64	
	1/128	1/32	1/32	1/8	1/64	1/128	
**Mean**	**90.4±1.4** a	**88.7±1.5** a	**85.4±3.1** a	**88±7.9** a	**90.9±2.7** a	**88.1±1.6** a	**8.6±5.3^b^**
60	1/128	1/128	1/128	1/32	1/128	1/128	n.d.
	1/128	1/128	1/64	1/16	1/1024	1/128	
	1/512	1/128	1/32	1/16	1/128	1/64	
	1/128	1/128	1/8	1/64	1/128	1/128	
	1/512	1/512	1/16	1/16	1/128	1/32	
	1/128	1/64	1/16	1/16	1/64	1/128	
**Mean**	**91.1±1.9** a	**89.6±2.5** a	**87.8±4.5** a	**85.3±5.6** a	**91.1±1.8** a	**89.5±0.7** a	**7.3±4.7^b^**

a The HI titer was expressed as the maximum dilution capable to completely inhibit the agglutination of human type O erythrocytes by RHDV. Each titer value corresponds to an individual animal. In the assay, a hyperimmune serum against RHDV (with an HI titer of 1/2048) was used as positive control. All animals were immunized at days 0 and 21. n.d.: hemagglutination inhibition titers not detected. ^b^The result is also expressed as the mean ± standard deviation of inhibition values obtained from individual animals in cELISA. Different letters within a row indicate statistical significant differences for *p*<0.05, according to the Kruskal-Wallis and Dunn’s tests.

### RHDV VLP were Able to Enhance the Immune Response to a Protective Peptide Fragment from Classical Swine Fever Virus (CSFV) E2 Envelope Protein

**Figure 5:**
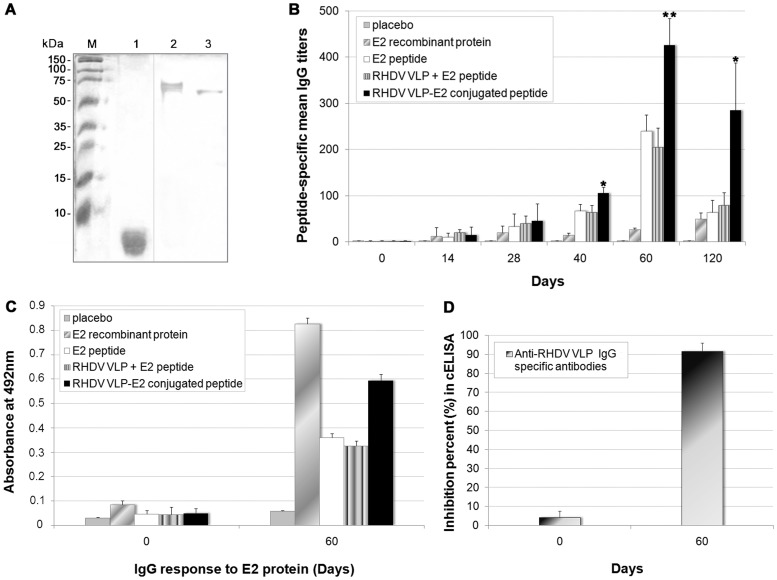
RHDV VLP as carriers for immunization against CSFV. (**A**) SDS-PAGE of conjugation of E2 peptide to the VLP. The 24 amino acids linear peptide from CSFV E2 glycoprotein was synthesized with purity over 95% and coupled to RHDV VLP. The gel was loaded with the synthetic E2 peptide (lane 1), VP1 conjugated to E2 peptide (lane 2) and unconjugated VP1 (lane 3). Numbers in the left and marker bands indicate the migration of standard molecular weight markers. (**B**) Antibody response generated in vaccinated pigs. Animals immunized twice with the E2 peptide conjugated to RHDV VLP showed the highest IgG peptide-specific titers, which were statistically superior to those obtained in the rest of the groups. Titers were expressed as the inverse of the maximum serum dilution having an OD superior to the negative control mean OD. (**C**) Antibodies able to react against the whole recombinant E2 protein were detected in the peptide immunized groups. (**D**) Antibodies with the capacity to compete for binding to RHDV were also detected by a modified cELISA in vaccinated pigs. For the analysis, an ANOVA was employed and mean titers were compared using the Newman-Keuls Multiple Comparison test. Statistical differences of the group immunized with the E2 peptide conjugated to VLP were indicated with one (*p*<0.05) or two (*p*<0.01) asterisks. Standard deviation bars are also shown.

Amino acid sequence alignment between a linear B-cell protective epitope from CSFV E2 glycoprotein (Shimen strain: AF092448) [42] and its homologue region in the Cuban highly pathogenic “Margarita” strain [43] was carried out. A peptide fragment was designed in which two amino acid changes (positions 697 and 713) were introduced within the 24 aa sequence, and also a cysteine residue placed at the C-terminal to direct coupling to VP1 multimers. The peptide fragment was synthesized with purity over 95% as corroborated by RP-HPLC and mass spectrometry. Conjugation of the E2 peptide to RHDV VLP was achieved with the use of M-maleimidopropionyl-N-hidroxysuccinimide esther. Conjugates were obtained as result of the reaction between the cysteine residue on the peptide and the maleimide-activated sites on VP1. An excess of peptide (5 mg) was utilized for conjugation to 5 mg of RHDV VLP. Once conjugation was accomplished, samples were subjected to dialysis in order to separate and remove possible unbound groups. Conjugation efficiency was visualized by comparison between unconjugated and conjugated proteins. Resulting conjugates showed a different pattern in SDS-PAGE with various protein bands migrating close to each other with sizes slightly superior than that of the unconjugated carrier (**Figure 5A**). Both VLP accessibility and peptide recognition after conjugation were corroborated with polyclonal antibodies obtained in mice against the peptide fragment and with the monoclonal antibody 1H8 directed to RHDV. Interestingly, RHDV VLP conjugates retained at least a partial binding affinity while the peptide was effectively recognized.

An experiment was conducted in rabbits and pigs with quite similar results in terms of specific IgG antibodies against the B-cell epitope coupled to RHDV VLP. Groups of animals were randomly created and immunized with 50 µg of conjugated VLP, 50 µg of peptide, both antigens co-formulated or 50 µg of an entire E2 recombinant protein [39]. No relevant uncommon lesions were observed at the sites of injection and animals maintained a healthy status during the experiment. An ELISA was developed to evaluate the peptide specific antibody responses after intramuscular immunizations administered two weeks apart. Animals immunized with the E2 peptide fragment conjugated to RHDV VLP showed the strongest IgG specific response that became statistically superior with respect to the rest of the groups after the second dose administered (**Figure 5B, C**). Pigs vaccinated with the peptide alone, animals injected with the soluble E2 peptide in addition to VLP, as well as the group immunized with the entire E2 recombinant protein, developed a similar though lower response against the peptide fragment. The peptide-specific response appeared to decline in the measurements performed by day 120, in which the differences between pigs immunized with the E2 peptide conjugated to VLP and the other groups were still marked. The humoral immune response was also evaluated by ELISA using the complete E2 homodimeric protein directly adsorbed onto the plates. The peptide-immunized groups showed IgG levels slightly inferior to those generated in animals vaccinated with the entire protein. Antibodies with the capacity to compete for binding to RHDV were also detected by a modified cELISA in vaccinated rabbits and pigs, as an additional indicator of multimeric structure preservation (**Figure 5D** **)**. Finally, a preliminary assessment for CSFV-specific neutralizing antibodies, according to [**39**], allowed detection of specific titers in three of the five pigs immunized with the RHDV VLP-E2 conjugated peptide after the second dose administered. These experiments showed the ability of stabilized RHDV VLP produced from yeast to present a viral B-cell epitope and enhance the specific immune response to it in a large animal model as swine.

## Discussion

It is known that several procedures result in stressing conditions for recombinant proteins subjected to purification, formulation or storage. Changes of pH, concentration of chaotropic agents, crossings of solubility thresholds, exposure to solid and air interfaces during concentration, centrifugation, filtration [44]–[46], as well as the use of excipients, temperature and time of storage, and freeze/thawing may promote structural changes leading to missfolding, unexpected protein-protein associations and aggregation.

In this work, we first analyzed the influence of pH during production of multimeric VP1. We considered previous laboratory evidence of process steps causing loss in VLP recoveries from solution due to aggregation events. Combined approaches have been also previously used to characterize multimeric antigens derived from this yeast strain [47]–[49]. As seen in the experiments, VLP in solution underwent changes that were dependent on pH. The patterns, retention times and recovery values suggested major stability in acidic to neutral pH, which was in part similar to findings described for Norwalk virus (NV) VLP analyzed under physical-chemical approaches [50]. NV VLP appeared more stable in the pH range 3–7 with a maximal stability at pH 4–5. The stability noticed for other calicivirus-like particles at acidic pH has been attributed in part to the capacity *in vivo* of some of these viruses to attach to the host gastrointestinal tract [50]–[52]. On this matter, a slight decrease in the final recovery at pH 3.0 was measured, which could be associated with the formation of chains or clusters, as seen by TEM, with a possible increased tendency to aggregate under stress. At pH 5.0 both the recovery and the colloidal stability were partially altered as reflected by insoluble aggregates visualized. This was probably the result of interactions between capsids that lost their native conformation due to environmental conditions, a fact that deserves further attention. At pH 8.0, the profile variation suggested the onset of dissociation in addition to partial aggregation. On the other hand, the association of the multimers in clusters or chains as visualized by TEM analysis resembled a quite similar phenomenon of particle adhesion described during large scale production of HCV core-like particles in this yeast strain [53]–[55]. Heterogeneity of the multimeric HBsAg and the formation of soluble aggregates during recombinant production and purification from *P. pastoris* were also previously documented [49]. At the end, the structure-dependent quantification of RHDV VLP confirmed that pH 4 or 7 were appropriate in terms of structure preservation and recovery without an increase in heterogeneity of the particles.

Process steps like concentration/diafiltration, temperature stresses and simulated storage provided useful data for production purposes. Volume concentration, buffer changing and sterile filtration constitute obligatory steps in order to attain the antigen concentration and sterility needed within the aqueous and oily-based formulations. However, phase changes as those provoked by storage at −20°C (freezing/thawing stress) or variations induced by long-term storage of the active raw material at 4°C could be avoided following a fermentation-to-adjuvantation uninterrupted production process as designed in our own production facilities for available vaccines [41], [26], [56].

With regard to the use of additives (if storage in solution for long periods is required), they must warrant the preservation of native conformational epitopes, the multimeric nature of the antigen and better recovery rates after storage. The use of stabilizers recognized as safe, like disaccharides sucrose and trehalose, or sorbitol and glycerol have shown in the past to be a useful strategy with inhibitory aggregation effects exerted by different mechanisms [57]. They are compounds known to be effective stabilizers of the structure of proteins [58]–[61]. Here, the capacity of additives to stabilize the VLP was concentration related and behaved similar at both pH assayed. It is valid to note that previous studies concluded that stabilizers of an antigen in solution give indeed formulations with improved thermal stability of the antigen in the adsorbed state [62], [63]. More recent findings on these yeast-derived VLP pointed as well to the utility of glucose 25% as inhibitor of aggregation under accelerated conditions at 60°C [64]. Based on the findings here described, it is also possible that other FDA-approved stabilizers could produce similar results.

A RHDV VLP-based vaccine is needed to be physically and thermally stable. Under these conditions antigen integrity within the formulation and maintenance of immunogenic capacity are highly relevant. Managing of veterinary vaccination has drawbacks like the necessity to deal with great numbers of animals along with laborious and stressing manipulation. In countries with subtropical or tropical climates there are also frequent cold-chain changes that result in thermal stresses. Here, the antigen integrity within the samples kept at 48°C was only slightly affected at the multimeric form level, thus generating a portion of disassembled (monomeric or dimeric) molecules. Even though no obvious changes were seen by TEM, heterogeneous populations must have been present, a fact that can be assessed by determination of diffusion coefficients and hydrodynamic sizes. Nevertheless, no differences were observed in the specific antibody response generated in the groups of rabbits injected with the stressed samples. The magnitude of the response reached was known to be sufficient to protect rabbits against RHDV [65]. As stability of RHDV VLP was only approached from a biochemical/antigenic perspective future work will deepens on stability under variable conditions using biophysical approaches and high resolution techniques.

The capacity to protect rabbits challenged with RHDV ensured the validity of the process from a clinical point of view. Also, the immunogenicity evidenced with formulations treated at 37 and 48°C agreed with the stability proved and supports the field use. The feasibility of using these multimers in a single dose regimen was also demonstrated. The ability to protect at even smaller doses has been previously assayed and found viable by authors that employed valuable expression systems though with less potential for production at large scale.

On this matter, single step purification by size exclusion chromatography using Sepharose CL4B appears a promising approach in terms of cost-effectiveness for large-scale production in detriment of ultrafiltration-based techniques. Results from the 10-L culture and purification processes are encouraging. Extrapolating the 10-L approach, and considering our own experience on cell growing experiments with this yeast strain and products derived from it [47], [48], [66], [25], [26] suggest that for instance, a 50-L culture will allow obtaining a final biomass of approximately 12.5 Kg wet weight, meaning 20.8 liters of cell material to disrupt (at 600 g/L) and about 44.6 liters of clarified disruption supernatant. Such quantities are also suitable to be processed in a 300 mm width×1000 mm height purification column. Under those conditions (column volume of 70686 mL), fifteen runs of 2827 mL each could be applied (4% charge) to render one peak per run containing approximately 860 mg of protein properly assembled. One day of purification may stand for nine runs, and one single process should produce over 257410 doses if formulated at 50 µg/mL. Moreover, at least three rounds could be conducted per packed column considering a 200-L culture. The latter would imply approximately 180 runs and a final production yield of around 2–2.5 million doses. An industrial process with related manufacturing steps and handling procedures is already established for the licensed Gavac^plus^™ veterinary vaccine, produced at CIGB facilities (HeberBiotec S.A.), aimed to fight ectoparasite infestations in cattle with prospective use in dogs [41], [67], [56]. At present, safety evaluation of RHDV VLP in rabbits, according to harmonized guidelines from VICH, has demonstrated both safety and efficacy under field conditions [68].

Expression levels of VP1, always over 300 mg per liter of culture, constitute an authentic advantage to consider this system for vaccine production even if the target is a small animal species. Recently, the potential of VLP, and specifically RHDV VLP obtained from insect cells, to induce humoral and cell-mediated immune responses against foreign epitopes incorporated by genetic fusion or chemical conjugation has been shown in successful experiments [31]–[33]. On this sense, it was recently reported the generation of specific responses in pigs by chimeric RHDV VLP (obtained from *Trichoplusia ni* cells) carrying a T-cell epitope of the 3A protein from foot-and-mouth disease virus (FMDV) [34]. In the present work, we used highly stabilized VLP obtained at elevated recovery rates as carriers against a relevant disease in pigs employing a B-cell epitope attached by chemical conjugation.

Our group has also developed a recombinant subunit vaccine candidate against CSFV that currently undergoes developmental stages [39], [69], [70]. This candidate is based on the expression of the E2 antigen in the milk of non-transgenic goats [70]. However, various groups have informed on the existence of B-cell neutralizing linear epitopes within the E2 sequence that protected pigs to variable extents against CSFV challenges [42], [71], [72]. Here, VP1 multimers were shown to successfully present one B-cell protective epitope to the immune system of pigs and enhance the IgG specific antibody response against this peptide. Chemical coupling has shown in the past to allow high peptide loads per VLP [31]. Our experiment compared the effect of the attached peptide with the immunogenicity of the peptide alone, the peptide co-administered with the VLP and with the response generated by the entire E2 recombinant protein. The presence of antibodies in animals immunized with the peptide fragment with the ability to react against the whole E2 recombinant protein was seen, to our knowledge, for the first time in pigs. Conversely, very recent reports have suggested the use of this fragment in combination with others in order to increase the protective efficacy [73], [74]. Other authors for instance, give attention to peptides covering amino acids 829 to 837 or 844 to 865 used under more immunogenic presentations [72], [73], [75]. Whether this fragment could be the most immunogenic or fully protective against CSFV will require challenge trials in which more experimental groups, fragment combinations and displaying strategies are considered. One interesting issue is that with the use of one of these epitopes, cell-mediated specific immune responses appeared to correlate with partial protection in pigs in the absence of neutralizing antibodies [74]. Thus, an ideal strategy would consider one or more B-cell (and T-cell) epitope for presentation. In summary, another element was provided to encourage the use of these multimers beyond the boundaries of vaccination against RHD, specifically for use in larger animals.

Many advantages and also limitations have been reviewed with regard to VLP modification and their use, including concerns on large-scale production, process control and optimization [76]–[79]. As VLP vaccines for humans are nowadays a reality [78], [29], the coupling or insertion of heterologous fragments with the intention of widen their practical value, along with technological improvements, must be a permanent research focus. Given the relevance of these trends, the establishment of an efficient platform to allow production/implementation of this kind of vaccine became an objective of this work. The results presented, and the knowledge gathered so far, support our endeavour to easily produce at large-scale safe, cost-effective RHDV VLP antigenically similar to RHDV, which may also be useful for additional purposes pertaining animal or human health. Future work will add to these results the parameters and requirements needed for recombinant vaccine production meant for use in human populations.
